# Relationship between pp65 antigenemia levels and real-time quantitative DNA PCR for Human Cytomegalovirus (HCMV) management in immunocompromised patients

**DOI:** 10.1186/1471-2334-7-138

**Published:** 2007-11-23

**Authors:** Elisabetta Cariani, Caterina P Pollara, Barbara Valloncini, Francesca Perandin, Carlo Bonfanti, Nino Manca

**Affiliations:** 1 Department of Experimental and Applied Medicine, Section of Microbiology, University of Brescia, and A.O. Spedali Civili, Brescia, Italy; 2 Laboratorio di Patologia Clinica, Ospedale Civile S. Agostino-Estense, via Giardini 1355, 414041 Baggiovara (MO), Italy

## Abstract

**Background:**

Quantitative real-time PCR assays, which are more rapid and practical than pp65 antigenemia determination, are progressively becoming the preferred method for monitoring Human Cytomegalovirus (HCMV) reactivation. However, the relationship between HCMV DNA and antigenemia levels is still under investigation. The aim of this study was to analyse the relationship between HCMV DNA and pp65 antigenemia levels in order to identify clinically useful threshold values for the management of patients.

**Methods:**

475 consecutive samples from 156 immunosuppressed patients were tested for HCMV by pp65 antigenemia and Real-time PCR assay.

**Results:**

136 out of 475 consecutive samples derived from 48 patients showed evidence of HCMV infection. HCMV DNA was detected in 106 samples, pp65 antigen in 3, and both markers in 27. pp65 antigen detection was associated with higher HCMV DNA levels. The cut-off HCMV DNA level that best predicted pp65 antigenemia in this series of samples was 11,500 copies/ml, but different threshold levels could be observed for specific groups of patients. HCMV disease was observed in 5 out of 48 patients with active HCMV infection. The presence of clinical symptoms was associated with positive pp65 and with higher antigenemia levels. Higher HCMV DNA load at the onset of viral replication was correlated to the development of clinical symptoms.

**Conclusion:**

Both pp65 antigenemia and HCMV DNA load can be useful for the prospective monitoring of immunocompromised subjects. Specific cut-off levels capable of triggering preemptive antiviral treatment should be determined in accordance to the type of test used and the characteristics of patients and prospectively validated.

## Background

Human Cytomegalovirus (HCMV) is the cause of lifelong latent infection in immune competent host. HCMV reactivation is a frequent event and represents a significant cause of morbidity and mortality in immunocompromised patients. The availability of diagnostic assays for the early identification of reactivated HCMV replication has considerably improved the clinical management of immunocompromised patients, thus reducing the risk of HCMV disease and allowing pre-emptive treatment as an alternative to universal prophylaxis. The determination of pp65 antigenemia has long been the reference test for monitoring HCMV reactivation [1-4], but it requires highly skilled interpretation and processing of fresh samples [5,6]. Furthermore, the evaluation of its results may be particularly difficult in leukopenic patients [7]. More recently, the quantitation of HCMV DNA by real-time PCR methods has been proposed as a convenient alternative approach, in order to overcome the drawbacks of antigenemia assays [7-17]. The exact relationship between HCMV DNA and antigenemia levels, however, is still under investigation. Studies addressing this issue would be instrumental in finding suitable threshold levels for the clinical interpretation of HCMV DNA results.

## Methods

### Patients

From June 2004 to March 2005, a total of 475 consecutive blood samples were received by our Laboratory for the simultaneous determination of pp65 antigenemia and quantitative HCMV DNA in plasma. The samples came from 156 patients (56 females, 100 males, mean age 44.4 ± 18.4 years). The median number of samples per patient was 8 (range: 6–28).

Patients included 75 (48%) kidney transplant (KT) recipients, 18 (11.5%) bone marrow/stem cell transplant (BMT/HSCT) recipients (including 16 pediatric and 2 adult patients), 34 (21.8%) patients with advanced human immunodeficiency virus (HIV) infection and 29 (18.5%) patients with haematological malignancies.

Active HCMV infection was defined as the detection of HCMV antigenemia and/or DNAemia. HCMV disease was defined as the association of active infection with clinical symptoms such as: unexplained fever (> 38°C), leukopenia (white blood cells < 3.5 × 10^9 ^/l) and/or thrombocytopenia (platelet count < 100 × 10^9^/l), gastrointestinal symptoms, artrhalgia, myalgia in KT recipients; fever, leukopenia, hepatitis, enteritis, retinitis or pneumonitis in BMT/HSCT recipients; colitis, oesophagitis, hepatitis, encephalitis or retinitis in patients with advanced HIV infection; fever and neutropenia (< 500 neutrophilis/ul) in haematological patients.

No antiviral prophylaxis was given to KT recipients, that received steroids (methylprednisolone 500 mg IV) before transplantation. In addition the patients received either an anticalcineurin agent or sirolimus. Intravenous ganciclovir or valganciclovir was administered when 50 or more pp65-antigen positive cells were detected. BMT/HSCT recipients underwent standard prophylaxis for bacterial and fungal infections, and received acyclovir 5 mg/kg body weight. Prophylaxis for HCMV infection was not administered. Intravenous ganciclovir (5 mg/kg of body weight every 12 h for 14 days, adjusted to renal function) was started when > 1 pp65-positive cells/200,000 PMNL were detected, or after two consecutive HCMV DNA-positive samples. The same criteria were used for pre-emptive treatment of haematological patients. Patients with advanced HIV infection had CD4 cell count < 100 × 10^6^/l and positive HCMV serology. They received antiretroviral therapy but no HCMV prophylaxis. Antiviral therapy was started when > 100 pp65 -antigen positive cells/200,000 PMNL and/or HCMV DNA load > 100,000 copies/ml was detected.

The donor/recipient serological status was determined before transplantation by a commercially available enzyme-linked immunosorbent assay (BEIA CMV IgG Quant, Bouty, Italy) according to the manufacturer's instructions.

All transplanted patients were monitored for the presence of active HCMV replication for 6 months after transplantation. Determination of HCMV DNA load and of pp65 antigenemia was carried out once (in the absence of HCMV reactivation) or twice a week (during HCMV active replication) until disappearance of antigenemia and/or DNAemia; subsequently, HCMV replication was monitored monthly. HIV-infected and haematological patients were monitored monthly during routine clinical controls. In the presence of clinical signs or symptoms suggestive of HCMV disease monitoring was carried out once a week.

### HCMV pp65 antigenemia assay

Twin blood samples were collected in citrate-anticoagulated tubes for pp65 antigenemia and quantitative real-time PCR. One sample was processed for antigenemia within 4 hours, and the other was stored for quantitative plasma DNA determination. The determination of HCMV antigenemia was carried out using a HCMV BriteTurbo kit (Lagitre U.S.A.) according to the manufacturer's instructions. The number of pp65-positive polymorphonuclear leukocytes (PMNL) was determined after staining with specific fluorescein isothiocyanate-labelled monoclonal antibodies, with results expressed as the number of positive cells per 200,000 PMNL.

### HCMV DNA quantitation

DNA extraction from plasma (200 μl) was performed using a QiaAmp DNA Mini Kit (Qiagen, Germany) according to the manufacturer's instructions. HCMV DNA quantitation was carried out by a commercially available real-time PCR test: TaqMan Q-CMV Real Time System (Cepheid, Nanogen Advanced Diagnostic, USA). This test is based on the simultaneous amplification of the HCMV MIEA (Major Immediate Early Antigen) gene (HCMV UL123-exon 4) and of internal control human beta globin gene DNA, that was added to each sample at a known concentration during specimen processing to monitor DNA extraction and recovery, amplification and detection. Specific TaqMan probes labelled with different reporter molecules (FAM/MGB-NFQ and VIC/MGB-NFQ) were used for target quantitation by interpolation of fluorescent signal intensity with a standard curve derived from the amplification of serially diluted HCMV DNA standards.

The assay was performed according to the manufacturer's recommendations. Briefly, DNA (5 μl) was added to the PCR mixture containing 5 mM MgCl2, 0.5 μM of each primer and probe and 0.025 U of uracyl-N-glycosylase (UNG). The PCR conditions were: decontamination at 50°C for 2 minutes, initial denaturation at 95°C for 10 minutes followed by 45 cycles of 15 sec at 95°C and 1 minute at 65°C. A plasmid containing UL123 gene diluted to give a standard curve of 2 log10 to 5 log 10 copies per reaction was used as a standard for quantification. PCR reactions were performed using an ABI Prism 7300 Sequence Detection System (PE Applied Biosystems). The linear interval of the assay's analytical sensitivity as assessed by the manufacturer was between 316 and 12.5 × 10^6 ^HCMV DNA copies/ml of plasma.

### Cost analysis

The costs of the assays were measured in terms of requirements for time, reagents or kits, and labour.

### Statistical analysis

HCMV DNA levels were compared by Mann-Whitney U test. The correlation between HCMV DNA and pp65 antigenemia was analysed using the Spearman correlation coefficient.

The differences between groups were analysed using the Chi-square test or the Fisher's exact test, as appropriate. A P value of < 0.05 was considered as statistically significant. Receiver-operating characteristic (ROC) curve analysis was carried out on the MedCalc statistical software, version 7.5.00.

## Results

### Analytical performance of the real-time PCR assay

To analyse the precision of the real-time PCR assay, inter- and intra-assay variations were calculated by testing three plasma pools containing samples with low (500–5,000 HCMV DNA copies/ml), intermediate (10,000–70,000 HCMV DNA copies/ml) and high (70,000–120,000 HCMV DNA copies/ml) viral load value. Intra- assay variation was evaluated by quantifying HCMV DNA load in triplicate for each plasma pool while inter-assay variation was calculated by analysing each plasma pool in four different assays.

The mean DNA loads (copies/ml) detected in the low, intermediate and high titre plasma pools, and intra-/inter-assay percent coefficients of variation (CV%) are shown in Table 1.

**Table 1:** Reproducibility of real-time PCR with low-, intermediate-, and high-titre plasma pools.

	**PLASMA POOLS**					
	
**PRECISION**	**Low-titre**		**Intermediate-titre**		**High-titre**	
	
	**Mean value (copies/ml)**	**CV%**	**Mean value (copies/ml)**	**CV%**	**Mean value (copies/ml)**	**CV%**
**Intra-assay**	3,162	27.8	50,119	23.5	100,000	21.2
**Inter-assay**	3,981	28.8	63,096	23.3	107,000	26.7

### Correlation between pp65 and HCMV DNA load

Of the 475 samples analysed, 339 (71.3%) were negative for both HCMV DNA and pp65 antigenemia. The remaining 136 samples, taken from 48 patients (20 females, 28 males, mean age 42.3 ± 17.8 years), were positive for HCMV DNA (106 samples, 78.7%), pp65 antigenemia (3 samples, 2.2%), or both (27 samples, 19.1%). HCMV assay results were discordant for 109 out of 475 samples (22.95%). Altogether, 51 episodes of active HCMV replication (defined by positive results of HCMV DNA and/or pp65 in at least two consecutive samples) were detected.

**Figure 1:**
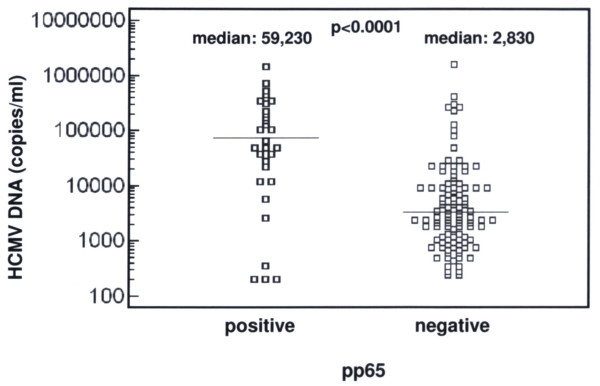
HCMV DNA levels in pp65-positive and -negative samples. The horizontal lines represent median HCMV DNA values in the two groups of samples.

The overall pp65 antigenemia values were statistically correlated to HCMV DNA levels (p < 0.0001). All samples containing detectable pp65 antigen had significantly higher HCMV DNA levels (median: 59,230 copies/ml; range: 0–1.47 × 10^6^) compared to pp65-negative samples (median: 2,830 copies/ml; range: 0–1.58 × 10^6^) (p < 0.0001) (Figure 1).

Significant differences were observed among median HCMV DNA levels in the different risk groups (Table 2). Samples from KT recipients contained significantly higher HCMV DNA levels compared to those derived from BMT/HSCT recipients (p < 0.0001), HIV-infected (p = 0.0056), and haematological patients (p = 0.0001).

**Table 2:** Detection of HCMV DNA and pp65 in different groups of patients

**Groups**	**n. positive patients (samples)**		**Median HCMV DNA copies/ml (range)**
		
	**HCMV DNA**	**pp65**	
**KT recipients**	24 (66)	5 (16)	17,020 (438-1.58 × 10^6^)
**BMT/HSCT recipients**	8 (44)	4 (12)	2,480 (316-3.1 × 10^5^)
**HIV**	10 (14)	2 (2)	2,615 (240-2 × 10^5^)
**HM**	6 (9)	0	1,100 (316-2,7 × 10^3^)

**Total**	48 (133)	11 (30)	5,841 (240-1.58 × 10^6^)

KT: kidney transplant; BMT/HSCT: bone marrow/stem cell transplant; HIV: patients with advanced human immunodeficiency virus infection; HM: patients with haematological malignancies.

The cut-off HCMV DNA level that best predicted positive pp65 antigenemia in the entire study population was identified by ROC curve analysis as 11,500 copies/ml (area under curve: 0.867; sensitivity: 88.9%; specificity: 79.2%). When the analysis was focused on single groups of patients, different threshold values between pp65-positive and -negative samples were found in KT recipients (cut-off: 24,000 copies/ml; area under curve: 0..888; sensitivity: 100%; specificity: 78%) compared to BM/HSCT recipients (cut-off: 9,960 copies/ml; area under curve: 0.605, sensitivity: 58.3%; specificity: 94.3%). The low number of pp65-positive samples in the remaining groups of patients (Table 2) did not allow for statistical analysis. To better define the cost-effectiveness of pp65 and of HCMV DNA in predicting HCMV reactivation and disease, we considered the cost and technical required time of each determination. The real-time PCR assay for HCMV DNA quantitation takes 180 minutes (DNA extraction included) with a total time for the production a results of five hours, whereas the pp65 antigenemia procedure requires 300 minutes for single test and a time for results of ten hours. The cost per sample with antigenemia and real-time PCR tests are Euro 10.31 and Euro 35.09, respectively.

### Clinical utility of pp65 and HCMV DNA load

HCMV disease occurred rarely as symptomatic infection was observed in 5 of 48 patients with active HCMV replication (10.4%). HCMV DNA was detected in all 48 patients whereas pp65 was detected in 11, including 5 with symptomatic infection (p < 0.001).

Among KT recipients (24 patients, 22 of which donor [D] positive [+]/recipient [R] +, 1 D+/R negative [-], 1 D-/R-) only one patient (D+/R-; 4.17%) developed symptomatic infection (fever and leukopenia) 44 days after transplantation. In this patient the diagnosis of HCMV infection was based on the simultaneous detection of pp65 and HCMV DNA. In the remaining 23 asymptomatic KT recipients, diagnosis of HCMV reactivation was made due to the detection of HCMV DNA with (4 cases) or without (20 cases) pp65.

HCMV disease symptoms, including fever, leukopenia, increased aminotransferase levels, gastritis and pneumonitis, were observed in 4 (66.6%) out of 8 BMT/HSCT D+/R+ recipients during the first two months [range : 20–59 days] after transplantation. HCMV DNA preceded the onset of symptoms and pp65 detection in 3 of 4 cases, whereas pp65 and HCMV DNA appeared simultaneously in the last patient. The remaining 4 asymptomatic BMT/HSCT recipients only showed low-level HCMV DNA in the absence of pp65. In the remaining patients asymptomatic HCMV reactivation was diagnosed by the detection of HCMV DNA alone (15 cases) or simultaneously with pp65 (1 case). Altogether, HCMV DNA appeared before antigenemia in 3 out of 5 patients developing symptoms and in none of 6 pp65-positive patients without symptoms of HCMV disease.

In symptomatic patients 29.1% of samples were positive for pp65 compared to 11.8% in asymptomatic patients (p < 0.001) (Table 3). Moreover, symptomatic infection was accompanied by higher pp65 levels (median: 30 cells/200,000 PMNL, range 3–100, vs median: 11.5 cells/200,000 PMNL, range 1–85, in asymptomatic patients; p = 0.029). ROC curve analysis showed that the best cut-off pp65 value predictive of symptomatic infection was 13 cells/200,000 PMNL (area under curve: 0.734; sensitivity: 81.2%, specificity: 57.1%). By contrast, median HCMV DNA levels did not differ significantly between symptomatic (7,500 copies/ml) and asymptomatic patients (4,580 copies/ml).

**Table 3:** Comparison of HCMV DNA and pp65 detection in 55 samples obtained from 5 symptomatic (with HCMV disease) and 119 samples from 43 asymptomatic (without HCMV disease) patients with active virus replication.

** VIRAL MARKER**			**TOTAL SAMPLES**
		
	**WITH HCMV DISEASE**	**WITHOUT HCMV DISEASE**	
**HCMV-DNA positive**	43	90	133
**HCMV-DNA negative**	12	29	41
**HCMV-pp65 antigen positive**	16	14	30
**HCMV-pp65 antigen negative**	39	105	144

Median HCMV DNA level in the first positive sample was 22,400 copies/ml (range: 1830–520,000) in the 5 symptomatic patients, and 2677 copies/ml (range: 280–413,820) in the 43 asymptomatic patients (p = 0.029). By contrast, the number of pp65 positive cells in the first positive sample did not differ significantly between symptomatic and asymptomatic patients (30 cells/200,000 PMNL, range 3–80, vs. 11 cells/200,000 PMNL, range 3–85; p = 0.17). A HCMV DNA load > 7880 copies/ml at first detection could predict the onset of symptoms with 80% sensitivity and 79.1% specificity (area under curve: 0.800).

HCMV infection responded to ganciclovir in all patients. The clinical monitoring of symptomatic patients during treatment revealed that pp65 became undetectable after 8–17 days (median: 12.5 days), whereas HCMV DNA decreased more slowly disappearing 7–105 days (median: 41 days) after the beginning of treatment. Similar results were observed among patients without clinical symptoms: pp65 (detected in 6) disappeared after 7–20 days (median: 14 days), whereas HCMV DNA persisted for 3 – 51 days (median: 33 days).

## Discussion

HCMV pp65 antigenemia is known to be clinically useful for the management of HCMV reactivation in immunocompromised patients [1-4]. Cut-off antigenemia levels have been proposed as an option to trigger treatment in different patient populations [18-20], which would reduce the incidence of HCMV-related disease.

Real-time PCR methods for HCMV DNA quantitation could offer a convenient alternative for monitoring HCMV replication which is less influenced by the quality of sample and more suited to standardization and automation, compared to antigen detection tests. In addition, real-time PCR requires a small volume of plasma (200 μl) compared to 3–5 ml of whole blood required for antigenemia, and can be done on neutropenic patients. Finally, despite being considerably cheaper, pp65 requires longer technical labour and longer time for the production of a result and the processing of fresh samples.

Although several studies have already addressed this issue [9,13,14,16,19-23], the correlation between antigenemia results and real-time PCR, as well as the clinical significance of both assays, requires further evaluation. When we analysed a series of consecutive samples referred to our laboratory, the sensitivity of the real-time PCR- based test in detecting HCMV infection was considerably higher than that of pp65 determination, although pp65 was detected in 3 samples with no evidence of HCMV DNA. These samples were consecutively derived from a single HSCT recipient after a symptomatic episode of HCMV reactivation with simultaneous detection of pp65 and DNA (data not shown).

Discrepancies between the two HCMV markers may be linked to the distinct viral components analysed here (protein vs. genome), the different districts considered (PMNL vs. plasma), the sensitivity threshold of the tests. The observation of pp65-positive, HCMV DNA-negative samples could be due to pp65 transfer from endothelial cells to adhering PMNL, in the absence of viral replication [13,24]. A significant correlation between pp65 antigenemia and HCMV DNA levels was demonstrated using a non-parametric test, despite the wide dispersion of HCMV DNA levels observed, in agreement with previous studies [13,14,20].

Prospective laboratory monitoring of patients allows for the timely adoption of therapeutic measures, thus reducing both the incidence and severity of HCMV disease in immunocompromised patients. Some studies describe an earlier rise in HCMV DNA levels, compared to antigenemia, in prospectively monitored patients [11,14,17], while the opposite observation was reported by others [24,25] In the present study both antigenemia and DNA were first detected together in 8 out of 11 pp65-positive cases. Furthermore antigenemia disappered earlier compared to HCMV DNA both in symptomatic and in asymptomatic patients, consistent with other reports [25].

HCMV DNA levels were compared between samples with/without evidence of pp65-positive cells, considering pp65 antigenemia > 0 as the lowest possible cut-off level for the prediction of active HCMV infection. Significantly higher HCMV DNA levels were associated with the presence of pp65 antigenemia, while the threshold HCMV DNA value for optimal discrimination of pp65-positive or -negative samples differed according to the risk group. This observation suggests that the level of immune suppression can influence the relationship between HCMV DNA replication and symptomatic HCMV disease. Accordingly, different cut-off HCMV DNA values for risk of disease were detected in solid organ and stem-cell transplant recipients [14]. The identification of discrepant threshold levels also suggests that the optimum cut-off value should be assessed specifically for each centre and assay [19].

HCMV disease was observed in only 5 transplant recipients (10.4% of patients showing HCMV reactivation), who developed symptomatic infection shortly after kidney transplantation (1 patient) or BM/HSC transplantation (4 pediatric patients). The detection of pp65 was associated with symptomatic infection, and significantly higher pp65 levels were observed in symptomatic patients. A cut-off antigenemia level of 13 positive cells/200,000 PMNL, comparable to previous results [21], allowed the best discrimination between symptomatic and asymptomatic patients. By contrast, both HCMV DNA detection and the circulating DNA load could not distinguish between symptomatic and asymptomatic patients. However, at onset of reactivation, HCMV DNA load was significantly higher in patients that developed disease, with best discrimination at about 8,000 copies/ml, a higher value compared to a previous result (2,000 copies/ml) obtained with a commercial quantitative PCR [21].

## Conclusion

In this study pp65 antigenemia, despite lower sensitivity for the diagnosis of HCMV active infection compared to HCMV DNA load, showed better qualitative and quantitative correlation with the presence of symptoms. Only at onset of active replication was the DNA load able to discriminate patients at risk of developing HCMV disease. On technical grounds however pp65 antigenemia, although less expensive, is often difficult to perform in leukopenic samples and suffers from major pitfalls such as labor-intensive manual procedure, requirement for immediate sample processing, subjective interpretation of results. Thus both tests can be useful for the prospective monitoring of immunocompromised subjects. Clinically relevant cut-off levels should be identified for specific groups of patients and assays, and prospectively validated for their predictive value over the development of clinical symptoms of infection.

## Competing interests

The author(s) declare that they have no competing interests.

## Authors' contributions

EC and CPP designed the study, collected clinical data, analyzed the results, and wrote the manuscript; BV carried out experimental work and collected laboratory data; FP and CB contributed to data collection and draft of the manuscript; and NM revised the manuscript. All authors read and accepted the final version of the manuscript.

## Pre-publication history

The pre-publication history for this paper can be accessed here:


